# An unusual presentation of pheochromocytoma accompanied by catecholamine‐induced cardiomyopathy

**DOI:** 10.1002/ehf2.15328

**Published:** 2025-07-04

**Authors:** Hugh O.J. Roberts, Alexandru Munteanu, Jonas E. Mertens

**Affiliations:** ^1^ Whangarei Hospital Whangarei New Zealand; ^2^ Department of Anaesthesiology and Intensive Care Medicine Campus Charité Mitte and Campus Virchow‐Klinikum, Charité – Universitätsmedizin Berlin Berlin Germany

**Keywords:** cardiomyopathy, case report, catecholamine, pheochromocytoma, takotsubo

## Introduction

Pheochromocytomas are catecholamine‐secreting tumours arising from chromaffin cells in the adrenal medulla. They are rare, with an annual incidence in Europe of 0.2 per 100 000 people.^1^ Diagnosis is challenging due to their rarity and non‐specific symptoms. Half are discovered incidentally on computed tomography (CT) or magnetic resonance imaging (MRI). These tumours are histologically indistinguishable from extra‐adrenal catecholamine‐secreting neoplasms, commonly referred to as paragangliomas. The majority of catecholamine‐secreting tumours occur sporadically; however, approximately 40% are associated with hereditary syndromes. Familial cases are more likely to present with bilateral pheochromocytomas or multifocal paragangliomas. All known familial syndromes associated with these tumours follow an autosomal dominant pattern of inheritance, including Von Hippel–Lindau (VHL) syndrome, multiple endocrine neoplasia type 2 (MEN2) and neurofibromatosis type 1 (NF1). The prevalence of pheochromocytoma in individuals with these syndromes is approximately 10%–20% for VHL, 50% for MEN2 and 2%–3% for NF1. Symptoms typically include at least two of the ‘classic triad’: headache, sweating and tachycardia.^2^ Hypertension is the most frequent symptom, although 10% of patients are normotensive.^1^ Rarely, pheochromocytoma is associated with cardiomyopathy attributed to catecholamine excess (catecholamine‐induced cardiomyopathy, CICM) that is similar to stress‐induced cardiomyopathy (also known as takotsubo syndrome, TTS).^3^ Twenty‐nine percent of pheochromocytomas are malignant, but the commonest causes of mortality are complications related to high circulating levels of catecholamines, including stroke, acute renal failure, ischaemic heart disease, arrhythmias, heart failure and pulmonary oedema.^4^, ^5^ Definitive management is by resection, requiring careful surgical technique and anaesthesia management to avoid inducing catecholamine release and subsequent uncontrolled hypertension. Principles of management of paragangliomas are the same, but anatomical location can make resection challenging. The most typical paraganglioma sites are the carotid body, jugular bulb, middle ear and vagus nerve, and lower cranial nerve deficits are frequent complications of surgery.^6^ Genetic testing is recommended for all individuals diagnosed with catecholamine‐secreting tumours and is typically performed following resection and histopathological confirmation. In cases where a hereditary syndrome is identified, genetic evaluation is also indicated for first‐degree relatives to facilitate early detection and management.

## Case report

**Figure 1:**
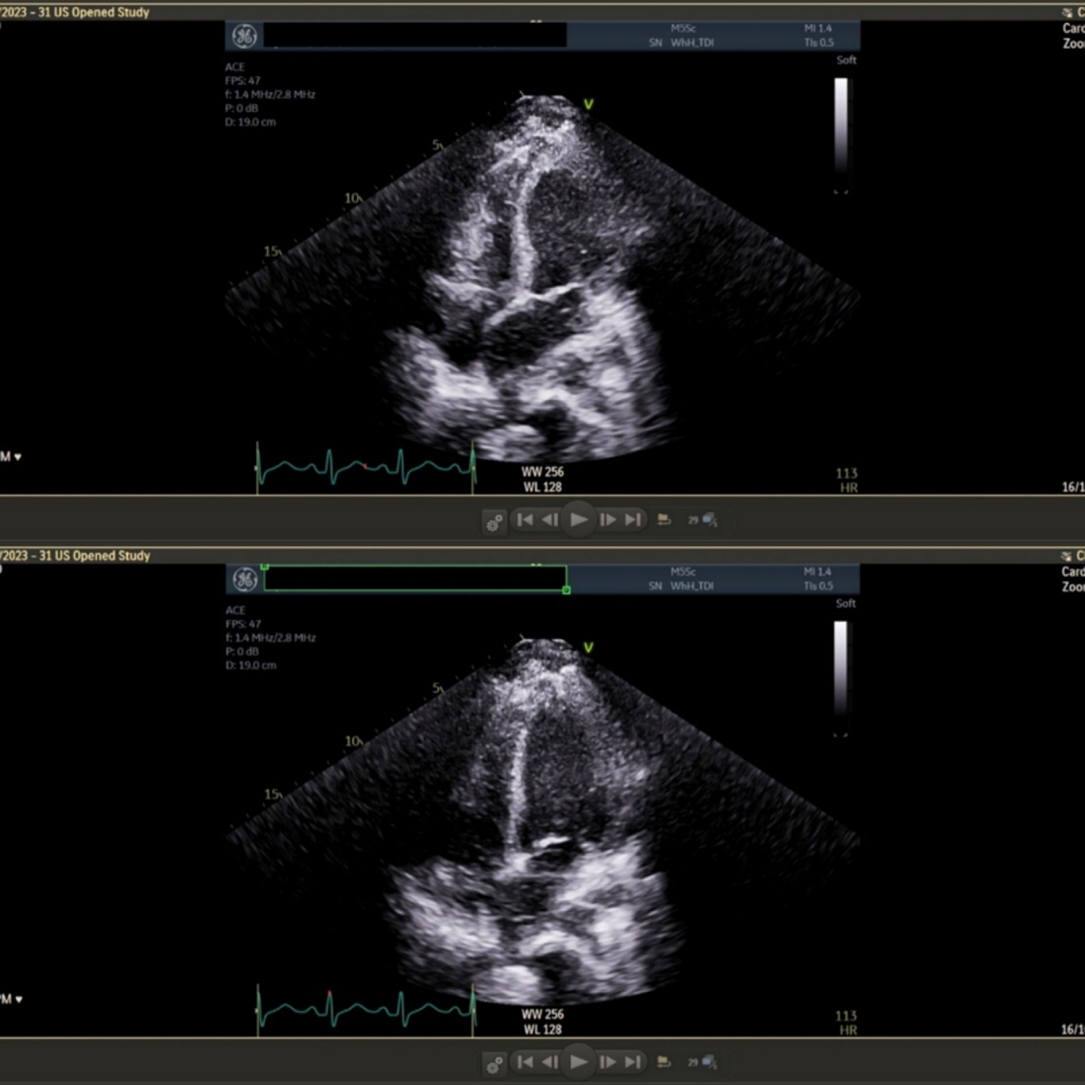
Transthoracic echocardiogram demonstrating mid‐segment hypokinesia of the ventricular septum during systole (top). An image of diastole (bottom) is included for comparison. A video recording of the investigation can be found in the Supporting information.

A 39‐year‐old female patient presented to the emergency department with intractable vomiting and a 2‐year history of recurrent shaking episodes with palpitations, tingling in her limbs and hot flushes. Migraine associated with nausea and vomiting often followed these episodes. Episodes had increased in frequency and now occurred daily, disrupting her life as a teacher. Despite review in a neurology clinic, no diagnosis had been established. In the emergency department, she was hypotensive, and bedside echocardiogram demonstrated global hypokinesia. Initial troponin was 543 ng/L, N‐terminal pro‐B‐type natriuretic peptide (NT‐proBNP) was 689 pmol/L and an electrocardiogram (ECG) demonstrated normal sinus rhythm with no ischaemic changes. She was admitted to intensive care for treatment with dobutamine and norepinephrine. Formal transthoracic echocardiogram showed left ventricle mid‐segment hypokinesia and a left ventricular ejection fraction of 33%, in keeping with mid‐ventricular variant TTS (see *Figure* 1). Coronary angiogram was normal.

Over 2 days on intensive care, the dobutamine and norepinephrine were weaned. The episodes of shaking, vomiting, and headache continued throughout the admission, and were associated with hypertension and ventricular ectopics. Once vasopressors and inotropes were discontinued, urine metanephrines were collected and were found to be grossly elevated. Phenoxybenzamine and bisoprolol were started for suspected pheochromocytoma. Prior to discharge, repeat echocardiogram showed normokinesis of all wall segments, and an improved ejection fraction of 62%. Outpatient CT confirmed a 55 mm adrenal pheochromocytoma, and laparoscopic adrenalectomy was performed 2 months later. Histology identified a clear resection margin and a low grading of adrenal pheochromocytoma and paraganglioma (GAPP) score, indicating a well differentiated type with a low risk of malignancy.^7^ Thirteen months post‐adrenalectomy, the patient reported no further episodes of shaking and associated symptoms or migraine since surgery. Plasma metanephrines were normal at 6 months and CT at 1 year showed no evidence of tumour recurrence.

## Discussion

### Diagnosis

Sporadic pheochromocytoma is a rare diagnosis with varied and non‐specific symptoms. The absence of hypertension, the commonest symptom, in 10% of patients complicates diagnosis. Hyperadrenergic spells, characterized by self‐limited episodes of palpitations, diaphoresis, headache, tremor and pallor are often the presenting complaint, but almost all patients seeking care with such spells will not have pheochromocytoma. Hence, misdiagnosis is common and these tumours usually rank low on the differentials list. This patient had been seen in neurology clinic for suspected migraines, but a diagnosis was yet to be established, and an encephalogram was awaited. The shaking episodes lasted between 5 and 30 min and interestingly, she reported that these initially affected her right limbs only, but later affected her whole body. Furthermore, trial of propranolol stopped the shaking episodes but caused intolerable nausea and had no effect on her headaches. Instead, she found that topiramate and ondansetron were effective. In clinic, her blood pressure was 125/61 mmHg. It is unclear if her symptoms were entirely due to the tumour or if the hyperadrenergic spells triggered migraines. While headache is a classic phaeochromocytoma symptom, nausea and vomiting are less typical but are common with migraine. Thirteen months post‐adrenalectomy, the patient reported that she had not experienced any further episodes of shaking, palpitations, paraesthesia, hot flushes, headaches, nausea and vomiting or headache since the surgery.

Diagnosis of pheochromocytoma is made by testing for metanephrine and normetanephrine, which are breakdown products of epinephrine and norepinephrine.^1^ Testing may be performed using urine or plasma samples. Consensus opinion from the First International Symposium on Pheochromocytoma, held in 2005, did not recommend one method over the other, with urine testing being more specific and plasma testing being more sensitive. In this case, urine testing showed grossly elevated metanephrine (27 300 pmol/L) and normetanephrine (28 700 pmol/L) levels (normal reference ranges <500 pmol/L and <800 pmol/L). Pheochromocytomas may secrete any one or a combination of epinephrine, norepinephrine and dopamine. Pure dopamine‐secreting tumours are rare and can be investigated by measuring urinary or plasma dopamine and homovanillic acid levels. Certain classes of drugs can interfere with results by causing increased urine and plasma metanephrines, including sympathomimetics, beta‐blockers, tricyclic antidepressants, venlafaxine, monoamine oxidase inhibitors and phenoxybenzamine.^8^ In this case, urine metanephrines required repeat testing as the first sample was collected while the patient was receiving a dobutamine infusion.

### Catecholamine‐induced cardiomyopathy and its overlap with takotsubo syndrome

CICM is a rare but well‐established complication of pheochromocytoma. It presents similar to TTS, mimicking a myocardial infarction with ST changes and/or T‐wave inversion, and sometimes with signs of cardiogenic shock including pulmonary oedema, arrhythmias, and even cardiac arrest.^3^ Both TTS and CICM result from high sympathetic stimulation, but one key difference is that TTS can occur without high plasma catecholamine levels.^9^

The reversible nature of CICM may be explained by the response of cardiomyocyte adrenoceptors to high epinephrine concentrations.^9^, ^10^ Counterintuitively, high epinephrine concentrations can cause negative inotropy by desensitization and downregulation of stimulatory cardiac β1 and β2 adrenoreceptors (b_1_AR and b_2_AR) by G protein‐couple receptor kinases (GRKs) and arrestins while increasing the activity of inhibitory cardiac β3 adrenoreceptors (b_3_AR). These are potentially protective mechanisms to prevent cardiomyocyte damage from overstimulation. Suppression of b_1_AR and b_2_AR causes a switch in cardiomyocyte cyclic AMP (cAMP) G‐protein receptor coupling from stimulatory G_s_‐proteins to inhibitory G_i_‐proteins. cAMP elevates intracellular calcium via protein kinase A‐mediated phosphorylation of calcium channels in the cell membrane and sarcoplasmic reticulum. The intracellular calcium increases inotropy via direct stimulation of stronger interactions between myosin fibres and actin filaments. Therefore, inhibition of cAMP production results in negative inotropy. Unlike b_1_AR and b_2_AR, b_3_AR are immune to desensitization by GRKs and arrestins. When b_1_AR and b_2_AR are suppressed, excess local catecholamines are diverted to b_3_AR. Activation of b_3_AR further decreases contractility by coupling to nitric oxide‐dependent pathway G_i_ proteins. In CICM, as catecholamine levels reduce (whether naturally or secondary to therapy), the switch back to G_s_‐proteins likely explains the rapid reversal of cardiomyopathy.^9^, ^10^

Despite limited data, which restrict our understanding of TTS and CICM, and their overlap in pheochromocytoma, several phenotypes have been described.^9^ The commonest variant is left ventricular apical hypokinesis, creating the characteristic ‘takotsubo’ (Japanese octopus trap) shape. Second commonest is mid‐variant, with basal and apex sparing, as seen in our case. Among the less prevalent phenotypes, basal variant has been associated with CICM secondary to pheochromocytoma.^9^ As more cases of CICM and TTS are reported, larger scale analyses should be able to identify and associate specific phenotypes and syndromes, aiding diagnosis and treatment.

To our knowledge, this is the first published case of a patient with CICM secondary to pheochromocytoma who presented with hypotension and suspected migraines.

## Conclusions

We describe an atypical presentation of pheochromocytoma, with CICM and hypotension. Pheochromocytoma diagnosis requires testing for metanephrines, but multiple drugs may interfere with these. CICM presents similarly to TTS, and several echocardiographic phenotypes have been described. More cases are required to determine the association of different phenotypes with different syndromes.

## Conflict of interest statement

The authors have no conflicts of interests or external funding to disclose.

## Supporting information


**Video S1.** Supporting Information
